# Stepped Transition to Employment and Postsecondary Education Success (STEPS) for Adolescents and Adults with Autism: Community Implementation Pilot Trial

**DOI:** 10.2196/70137

**Published:** 2025-08-29

**Authors:** Susan W White, Alexis M Brewe, Nicole Powell

**Affiliations:** 1 Department of Psychology, Center for Youth Development and Intervention, University of Alabama, 200 Hackberry Lane Suite 101, Tuscaloosa, AL, 35487, United States, 1 205-348-1967; 2 TEACCH Autism Program, Department of Psychiatry, University of North Carolina, Chapel Hill, NC, United States

**Keywords:** autism, adult, transition, intervention, community

## Abstract

**Background:**

Programming to optimize successful transition into adulthood and build skills for independence is consistent with the goal of improving autonomous living among adults with autism, which is a top stakeholder-identified priority. There has been surprisingly little research; however, on structured curricula targeting transition into adulthood.

**Objective:**

This formative community pilot trial of Stepped Transition to Employment and Postsecondary Education Success (STEPS) was designed to test feasibility and effectiveness as implemented by community-based providers and, secondarily, to identify factors that affect implementation.

**Methods:**

This was a 2-phase study. Phase 1 involved engagement with a group of community stakeholders to identify factors likely to influence implementation of STEPS. Phase 2 involved an open pilot trial of STEPS. In the Hybrid Type 1 trial, 24 adolescents and young adults with autism received STEPS in their communities at a local agency unaffiliated with the research study.

**Results:**

Based on stakeholder input (Phase 1), several adjustments were made to the program before implementation (eg, increased attention to building client motivation and clarification of the role of caregivers). Stakeholders and providers indicated that STEPS could be successfully delivered and adopted in the community. From the pilot (Phase 2), results indicate feasibility of study procedures and intervention implementation, supporting future larger-scale implementation. Satisfaction (eg, how helpful and beneficial) with the program was reported as moderate or higher by the participants, and the only 2 drops occurred before the program start. Caregiver-rated transition readiness significantly increased from baseline to end point (*P*<.001), as well as some domains of functional independence (finance management, self-care, and engagement in the community; all *P*<.05). Employment and education status at the end point did not yield a clear pattern indicating a positive or negative impact of the program.

**Conclusions:**

This pilot study supports the feasibility, acceptability, and effectiveness of STEPS as delivered by community providers.

## Introduction

Autism incurs an estimated lifetime per capita societal cost of US $3.2 million, and data suggest that lost productivity and adult care needs are the largest contributors [1]. As of 2020, adults with autism accounted for 35% of the overall autism service use sector; based on changes in rates of diagnosis during childhood, models project that adults with autism will constitute 71% of the cost of care by the year 2060 [2]. Consistent with these projections, stakeholder-identified research priorities place efforts to build skills necessary for adulthood, including seeking employment, and to improve services to support independent living among adults with autism as a top priority [3,4], although addressing lifespan issues like the transition to and supporting needs in adulthood has not historically been a highly active research area [5,6].

Adolescents and adults with autism experience lower subjective quality of life than do age- and cognitive ability-matched peers without autism [7,8], and they face challenges with living independently [9,10]. The majority of adults with autism are not consistently employed, and those who do secure employment are often underemployed, unable to support themselves financially or not working full-time [11,12]. In fact, paid employment has been linked to improved self-esteem and well-being for adults with autism [13]. Furthermore, young adults also experience elevated rates of dysphoric symptoms, including feelings that they do not belong or are a burden to their caregivers, depression, and suicidal ideation [14-16]. Transition-focused approaches serve to bridge the gap between childhood services and the services needed once an individual with autism leaves school, starts working, or requires different supports as they enter adulthood.

Transition planning for adolescents and adults with autism is frequently cited as lacking needed supports and skill-building [17,18], which is exacerbated by the “service cliff” of incredibly limited services and supports available to people with autism and their caregivers once adolescents with autism leave the K-12 school system [19]. The most commonly identified needs and challenges faced by emerging adults on the spectrum are in the areas of interpersonal competence, ability to manage competing demands, self-advocacy, and poor self- regulation [20,21]. Consistent with an experimental therapeutics framework [22], in which proximal processes that maintain the behavioral or clinical problem are targeted in order to bring about downstream changes in behavior or functioning, the Stepped Transition to Employment and Postsecondary Education Success (STEPS) program targets these domains in order to improve preparedness for adulthood among emerging adults with autism [23]. In a previous randomized controlled trial (RCT) with 59 adolescents and adults with autism, conducted in the lab where the program was developed, STEPS participants demonstrated significant improvement in transition readiness, compared with a “transition services as usual” control group, and this improvement was predicted by self-determination. Furthermore, STEPS was highly acceptable to families, feasible to implement with fidelity, and resulted in significantly increased readiness for transition, operationalized as age-typical attitudes and behaviors related to education, social interaction, and functional independence [24]. A recent small pilot of STEPS (n=12 individuals with autism) housed within a community-based nonprofit organization further illustrated high feasibility and acceptability and some individual-level improvement in transition readiness and self-efficacy [25]. Although more community-focused than the original RCT, this latest pilot study focused on additional “proof of concept” of STEPS to address individualized participant goals in employment, education, and daily living, as well as consideration of challenges to future implementation [25]. The important next step is to begin testing community implementation, while continuing to evaluate STEPS’ feasibility and clinical impact.

The goal of the present, formative study is to demonstrate feasibility, acceptability, and preliminary impact of community-implemented STEPS on independence and readiness for adult transition in a sample of teens and young adults with autism. This is in line with the guidelines for reporting of nonrandomized pilot studies [26]. Using a hybrid type 1 open trial design [27], we primarily assessed the feasibility, acceptability, and effectiveness of STEPS, with the secondary goal to assess factors that influence implementation, consistent with the Consolidated Framework for Implementation Research (CFIR) [28]. The CFIR is a widely used implementation science framework that helps to elucidate barriers and facilitators to implementation across 5 domains: innovation (eg, the new program), the outer setting (eg, the state or broader system), the inner setting (eg, the delivery agency), individuals (eg, characteristics of providers), and the implementation process (eg, how to plan and deliver the program) [28]. Hybrid trials, which simultaneously measure impact and implementation processes in the target community (rather than in a more controlled lab environment), may reduce the research to practice gap and hasten dissemination.

## Methods

### Overview

This was a 2-phase study. The goal of Phase 1 was to identify factors that would likely influence effectiveness, adoption, implementation, and eventual sustained use of STEPS. An implementation workgroup comprising community partner agency representatives and other stakeholders in the region (families, educators, and mental health professionals) met 3 times, before the trial’s launch, to elicit stakeholder thoughts and experiences relevant to the goal of optimizing program sustainability within community settings. Meetings were conducted over Zoom (Zoom Video Communications, Inc), each lasting 1 hour during which the research team presented informational slides, introduced the topics for discussion, and explained the relevance of the topics to the project. Meeting discussions included the full group and were led by the first author, covering implementation-related topics, guided by CFIR constructs. These included the “innovation” (eg, advantages and adaptability of STEPS), “outer setting” (eg, partnerships, policies, and financing), “inner setting” (compatibility and available resources), and “individuals” domains (eg, innovation deliverers). Meeting 1 focused on anticipated challenges in implementing a program targeting adult transition; meeting 2 addressed access to programming through schools and agencies, and ensuring program implementation fidelity; and meeting 3 covered engaging community champions, training needs for clinicians, and reaching underserved communities. Phase 2 involved the implementation pilot of STEPS in the community, which included 5 nodal assessments at eligibility, baseline, 7-week midpoint, end point at week 14, and follow-up 6 months after end point.

### Sample

Phase 1 individuals were recruited from the partnering agency’s network of professional connections, and the agency’s director facilitated their contact with the research team. Phase 1 participants were drawn from the study’s target location (predominantly rural area of the southeastern United States) and were recruited based on their knowledge and experience with regional autism services for adolescents and adults. Phase 1 participants provided informed consent at the time of enrollment.

Eligibility criteria for participation in Phase 2 included a previous autism diagnosis, confirmed by participants and their caregivers, and sufficient verbal skills to participate in STEPS, based on caregiver reports during the eligibility phone screening. There was no IQ cut-off to participate. Approximately 20% of the sample had a caregiver-reported previous diagnosis of intellectual disability. Participants were recruited from predominantly rural areas in the southern United States, using the partnering agency’s website and client list (see below for more information on the agency partners), supplemented by local events (eg, autism walks) and social media, and participants included both existing and new clients. Participants received general information about the study through personal contact with the partnering agency’s director or affiliated clinicians. If interested, the participant or caregiver completed a brief phone screen with the project coordinator. If eligible, the participant was assigned to one of the participating clinicians, according to a pre-established randomization process. When the provider had room in their caseload, they signaled the project coordinator, who facilitated scheduling. Phase 2 participants (TAY and caregivers) provided informed consent at the time of enrollment.

The partnering agency delivering STEPS was a community mental health clinic that accepted both public and private insurance. They did not specialize in autism, although some of the providers had previous clients with autism and served a diverse clientele. Participating providers were recruited from the partnering agency’s affiliated clinicians. Agency providers all had doctoral degrees (PsyD-2; PhD-2) and included 2 males and 2 females. The agency director facilitated initial contact between the providers and the research team, and coordinated online and in-person meetings in which the details of the study were explained and training in STEPS was provided (see below). All providers completed informed consent before their participation.

### STEPS Program

Described in detail elsewhere [23], STEPS is a modular cognitive-behavioral curriculum comprised of 12 content modules, meant to be delivered over a series of approximately 12 weekly sessions, one-on-one with a client. Informed by research and stakeholder input, STEPS was developed to facilitate the successful transition to adulthood among adolescents and young adults with autism by promoting essential skills for adult autonomy, including self-knowledge, self-determination, and self-regulation. In addition to the individual sessions, STEPS includes in vivo skills practice opportunities (termed “immersion”). Of note, in this trial, participants had one immersion during the program; based on consumer feedback, STEPS now includes 2 immersion sessions. The providers were trained during a full-day on-site workshop, including didactic presentation and role-play, before the start of the trial. They also participated in weekly supervision meetings with the lead author, via videoconference, throughout the trial. During these meetings, real-time program adaptations for clients were discussed, as well as troubleshooting to optimize applicability and personalization. This integrated training approach is similar to that used in other effectiveness trials with community agencies [29]. The qualitative feedback we will receive from providers will boost readiness for broad dissemination in the community.

### Measures

The following measures were used in Phase 2 only. Consistent with previous research on metrics of feasibility and acceptability of interventions in clinical trials [30,31], feasibility of STEPS (ie, how doable the intervention was in the community) was operationalized as high fidelity to the STEPS manual, as reported by providers and confirmed by an independent rater; at least moderate provider-reported overall feasibility; and at least moderate client-reported therapeutic alliance. These aspects of treatment feasibility were selected to measure both providers’ beliefs and behavior in delivering STEPS appropriately (ie, reported feasibility and fidelity in comparison to observer-rated fidelity), as well as whether delivery of all treatment objectives could still result in a good therapeutic relationship, given research demonstrating that feasibility consists of multiple components [30,32]. Acceptability of STEPS (ie, consumer and provider response to the intervention) was operationalized as low attrition, at least moderate provider-reported overall acceptability and intervention appropriateness, and at least moderate client- and caregiver-reported satisfaction ratings. These metrics were chosen to reflect clients’, caregivers’, and providers’ beliefs about STEPS following the trial, as well as behavioral data on whether clients decided to complete STEPS [31]. Implementation was assessed via qualitative feedback from providers. Consistent with the conceptual framework of STEPS (ie, targeting self-knowledge, self-determination, and self-regulation to exert sustained change in readiness for transition to adulthood and independent living skills), our primary outcome measures assess transition readiness and functional adult living skills.

### Client- and Caregiver-Completed Measures

American Institutes for Research–Self-Determination Scale (AIR-SD) assesses opportunity (ie, 12 items) and capacity (ie, 12 items) to choose activities and behave in goal-directed ways, key facets of self-determination. Participants completed the full self-report version, whereas caregivers completed the proxy report, which consisted of the full AIR-SD minus 6 capacity questions that measured their adult child’s internal perceptions and knowledge. We adapted the opportunity questions about school to include school, work, and other environments participants are regularly in outside of their home to more accurately reflect the age and goals of our sample. The AIR-SD is strong psychometrically (*α*=.95) and the 2-factor structure has been replicated in autism [33,34]. It is sensitive to treatment change, including in autism and in the original STEPS trial [35-37]. In the current sample, internal consistency was good for self-report (*α* ranged from .94-.96). Caregiver-reported capacity at end point had lower internal consistency (*α*=.45); otherwise, internal consistency was also good for caregiver reports (*α* ranged from .81-.92). The AIR-SD Opportunity and Capacity scores were used as a measure of SD. The AIR-SD was completed by participants and their caregivers at baseline, the 7-week midpoint, and the end point.

Emotion Dysregulation Inventory (EDI) [38] involves caregiver completion of the Reactivity-Short Form (7 items) and EDI Dysphoria (6 items), and participants completed an alpha version of the self-report EDI that consisted of the caregiver-report form reworded to person-first language. Both forms were used as a facet of self-regulation, in addition to the General Self-Efficacy Scale (GSES; described below), and assess the intensity and frequency of emotion dysregulation in an individual over the past 7 days [38]. The EDI captures 2 domains of dysregulation: reactivity, which is characterized by rapidly escalating negative emotions, and dysphoria, which is characterized by low positive affect or a motivation and a general sense of unease. The items are rated on a Likert scale, with higher scores indicating more emotion dysregulation. Participants and their caregivers completed the EDI at baseline, 7-week midpoint, and end point. The *t*-score conversions were completed based on norms generated from a sample of 1755 caregivers of youths with autism (ie, “autism norms”); self-report scores were also converted based on proxy report norms given the use of the alpha version of the self-report scale. The EDI has shown good construct validity and sensitivity to change [38]. Internal consistency ranged from .86-.97 for self-report and .59-.97 for caregiver-report.

General Self-Efficacy Scale (GSES) [39] is a 10-item form completed by participants and their caregivers at baseline, midpoint, and end point. It measures participants’ self-efficacy, or their belief in their ability to deal with stressful situations effectively. Higher scores reflect increased self-efficacy. The GSES has shown strong internal consistency and convergent validity [40,41]. Internal consistency in the current study was similarly good for self- and caregiver-report (*a* ranged from .79 to .98). The GSES Total Score was used as our measure of self-regulation.

Integrative Self-Knowledge Scale (ISKS) [42] is a 13-item self-report measure of both experiential and reflective self-knowledge, or understanding one’s experience over development (past, present, and future) in relation to one’s goals and desired outcomes. Higher scores reflect increased self-knowledge. The ISKS has strong internal consistency and convergent and discriminant validity [42]. Internal consistency in the current sample was also good (*α* ranged from .82-.92). The ISKS total score was used to measure self-knowledge. The ISKS was completed by participants at baseline, the 7-week midpoint, and the end point.

Program Satisfaction Scale (PSS) is a self- and caregiver-report form adapted from the Consumer Satisfaction Survey [43] to measure satisfaction with STEPS (ie, intervention acceptability). The PSS assesses satisfaction on several metrics, including helpfulness, relevancy of content and materials, satisfaction with participants’ progress, acceptability of format, and likelihood of referring another person to STEPS. Satisfaction with STEPS was defined as having an average PSS score of >3, which indicates moderate helpfulness, impact, and acceptability. On the PSS, scaling is as follows: 1=no or not at all; 3=moderate or somewhat; 5=very (helpful, beneficial, satisfied). The PSS was completed by participants and their caregivers at end point only.

Rehabilitation success is a measure that captures participants’ engagement in employment and education. Caregivers completed the form at baseline, end point, and 6-month follow-up by indicating yes or no about whether their adult or adolescent with autism was employed and engaged in educational or training programs (including high school and postsecondary education) at each time point.

Relationships, Employment, Autonomy, and Life Satisfaction (REALS) [44] is a compilation of scales that measure multiple facets of independence and adult life for adults with autism and adults with intellectual disability (ID). It has a self-report and proxy-report form. The REALS measures support, or the level of support an adult with autism needs to perform certain tasks, and frequency, which is how often the participant engages in those tasks. The REALS provides theta scores for support and frequency in the domains of social activity, work readiness, work performance, home care, self-care, leisure, mobility, finances, and health (including sleep, diet, and exercise; proxy report only). The self-report form also includes three satisfaction scales on social activity satisfaction, autonomy satisfaction, and work or school satisfaction. REALS psychometric testing is currently ongoing; in the current study, internal consistency was adequate for self-report (*a* ranged from .70-.98) and ranged from weak to strong for caregiver-report (*a* ranged from .47-.95). The REALS was used as a primary outcome measure. The REALS was completed by participants and caregivers at baseline, end point, and 6-month follow-up.

Seven Component Self-Determination Skills Survey (SCSDSS) [45] is a self- and caregiver-report of self-determination. The form provides total scores for performance and perceived importance of 7 skills involved in self-determination including choice-making, decision-making, goal setting, problem solving, self-advocacy and leadership, self-awareness and self-knowledge, and self-management. The SCSDSS also includes a 6-item version of the AIR Self-Determination Scale [46], which measures overall self-determination. Internal consistency was adequate for ratings of importance (*a* ranged from .70-.90), performance (*a* ranged from .72-.90), and overall self-determination (*a* ranged from .69-.90) across both caregiver- and self-report. All 3 total scores (importance, performance, and overall self-determination) at baseline, 7-week midpoint, and end point were used to examine change in self-determination as a mechanism of STEPS.

Transition Readiness Scale (TRS) [47] is a 30-item self- and caregiver-report measure of transition readiness. It was initially developed for the original STEPS RCT to measure readiness to enter college; however, it was adapted to measure general readiness for the transition to adulthood, including meeting one’s goals in postsecondary education or employment. Higher scores reflect more transition readiness. The TRS has demonstrated strong internal consistency, good item-level analyses, and discriminant and concurrent validity [47,48]. In the current study, internal consistency was good for both self- and caregiver-report (*a* ranged from .76-.91). The TRS Total Score was a primary outcome measure. The TRS was completed by participants and caregivers at baseline, 7-week midpoint, and end point.

Working Alliance Inventory–Short Revised, client version (WAI-SR) is a 12-item self-report measure of participants’ experience of the client-therapist alliance (ie, a metric of intervention feasibility). The items cover 3 aspects of working alliance, including goal (ie, agreement on clients’ presenting problems and goals for therapy), task (ie, agreement on how to achieve goals and willingness to work together toward those goals), and bond (ie, the affective relationship between client and therapist). Higher scores reflect stronger alliance. The WAI-SR has strong internal consistency and construct validity with other alliance measures [48,49]. Internal consistency was high in the current sample (*α*=.96). The WAI-SR was completed by participants at the 7-week midpoint only.

### Provider-Completed Measures

Session fidelity ratings were completed by providers following each STEPS session to capture providers’ adherence to the manual in delivering session objectives (a metric of treatment feasibility). Therapists reported whether or not each session objective was met. Therapist-reported fidelity was also confirmed by independent raters (i.e, trained research assistants) who co-coded 10% of all STEPS sessions for fidelity to the manual. Independent raters were trained to rate session fidelity by receiving an overview of STEPS, including an explanation of treatment objectives and practice coding sessions. To establish reliability, independent raters coded randomly selected session videotapes until they achieved exact agreement across 3 consecutive videos with the study authors. After reliability was achieved, the independent raters co-coded a randomly selected 10% sample of the total videotapes for each participant.

Implementation interviews were completed with each provider at the end of the trial to collect their feedback on implementation barriers and facilitators, based on their experience in delivering STEPS. Each one-on-one interview was brief (< 1 hour) and consisted of the same set of 21 questions covering each of the CFIR domains: innovation, inner setting, outer setting, individual domain, and Implementation Process [28]. After introducing each domain, a series of questions related to both hindrances and facilitators to the implementation of the program within that domain were posed. Examples of interview questions include “To what degree did client motivation affect use?”; ”What funding considerations need to be thought of?”; and “What provider characteristics or skills are important?”.

Implementation surveys included the Feasibility of Implementation Measure (FIM), Acceptability of Implementation Measure (AIM), and Intervention Appropriateness Measure (IAM) [50]. These 3 measures, comprised of 4 items each, are rated by providers to assess their perceptions of acceptability, appropriateness, and feasibility of adopting STEPS. The implementation surveys all demonstrate strong internal consistency and sensitivity to change [50]. Providers completed the implementation surveys immediately following completion of STEPS training and following completion of all STEPS cases for the trial. A score of ≥3 reflects moderate acceptability, appropriateness, and feasibility.

### Community Involvement

Phase 1 included several people with autism and advocates in the community (e.g., parents) who attended and contributed to the implementation workgroups (described above), sharing their personal experiences, thoughts, and opinions about services. In addition, the development of the STEPS program relied heavily on input from the autistic community [51]. To ensure scalability of the program and adoption in community agencies, providers and leadership from our partner agency were also included in Phase 1. This was critical for considerations such as training, billing, and provider qualifications.

### Data Analysis

The Phase 1 workgroup meetings, designed to ensure successful delivery of the program in Phase 2, were all recorded and transcribed to allow for thorough and accurate review. Given the exploratory nature of Phase 1 and the implementation interviews, as well as the fairly small samples, a structured analytic approach [52] was not deemed necessary. The lead author, who has previously led such qualitative endeavors, led the workgroups and conducted the interviews. An informal qualitative content analysis approach was used for qualitative examination of content [53]. Specifically, for each meeting, participants’ responses were individually examined, categorized according to the emergent topic areas (see results below), and considered collectively to summarize the results.

Phase 2 quantitative data were analyzed using SPSS (version 29.0; IBM Corp). Before any analyses, the data were cleaned, checked for missing data, and checked for assumptions. The amount of missing data varied across participants. Out of the 24 participants enrolled in the study, only 2 participants and 3 caregivers completed all data at every study time point. The highest missing data were found at the end point (23% of completers and 27% of their caregivers missing at least some data) and 6-month follow-up (59% of completers and 36% of their caregivers missing some or all their data). Preliminary analyses were conducted to examine if there were demographic differences in participants and caregivers who completed the end point versus not. There were no significant differences in demographics between those who did and those who did not complete the end point.

Descriptive analyses of all variables were run to characterize Phase 2 participants. Frequency and descriptive analyses were used to examine metrics of acceptability (ie, attrition rate, program satisfaction ratings, and providers’ ratings of acceptability and appropriateness) and feasibility (ie, provider- and observer-rated fidelity to the manual, provider-reported overall feasibility, and client-reported therapeutic alliance). Qualitative responses from interviews with providers during Phase 2 were informally reviewed for common themes and grouped under the CFIR domains to consider implementation factors. Given there were only four providers, a more rigorous approach such as thematic analysis is not viable. Descriptive analyses were also used to characterize outcomes on the Rehabilitation Success form. Clinical impact was tested in two ways. Given our small sample size and the high amount of missing data for participants across time points, we used paired samples *t* tests to examine statistical change in main outcomes (TRS, REALS) from (1) baseline to end point and (2) baseline to 6-month follow-up. Similarly, we examined change in mechanisms (ISKS, AIR-SD, SCSDSS, GSES, and EDI) from (1) baseline to 7-week midpoint and (2) baseline to end point. A significance threshold of *P*=.05 was used, without correction, given the pilot nature of the trial. We also examined clinically significant change, operationalized as movement of >0.5SD on the TRS from baseline to end point. Change was examined in both directions to identify positive and negative responders to STEPS.

### Ethical Considerations

This study received full ethics review board approval from the University of Alabama Institutional Review Board (IRB Protocol numbers: 21-12-5245; 21-12-5231), and all participants and caregivers provided written informed consent, with ample time to ensure all questions were answered, before any data collection. All data were deidentified to protect confidentiality. Participants received US$25 per assessment (5 in total; up to US$125 for full participation in the assessments). There was no payment, nor cost incurred, for receiving the program.

## Results

### Phase 1

Phase 1 participants comprised 16 individuals (4 male, 12 female), including parents (2), a young adult with autism (1), administrators at agencies serving individuals with autism (5), school system administrators (3), a university support services representative (1), and mental health providers (4). Topics explored in the stakeholder meetings included identification of potential implementation challenges (“Some [autistic adolescents and adults with autism] may not see the need to do the program, even though we see they need it, and because they’re adults, they choose to not want to”), reaching and retaining families in the program (“…many drop off our radar and we don’t have the resources to follow up with them”), training issues (“One of the challenges is the training of the clinicians to make sure that you reach that reliability, which is important. So, you don’t have one clinician doing one thing and another doing another”), reaching underserved and disadvantaged populations (“a number of these kids have Medicaid services…oftentimes those [care management] companies will send out bulletins to providers and to members so that might be something to consider”), and funding issues (“… in the adult service world, funding is so limited, and you have to be creative. It is a large barrier”). Based on stakeholder input, several adjustments were made to the STEPS program before implementation. The manual was edited to encourage incorporation of special interests to increase participant motivation, more visuals were incorporated into program materials and handouts, notes and tips were added to assist clinicians in anticipating common implementation issues, and more diverse examples were provided for immersion activities. An introductory section was also added to clarify the role of caregivers in the program (ie, that the “client” is the teen or adult with autism, with caregivers in a supportive, but peripheral, role). Please see Table 1 for additional quotations from Phase 1 meetings.

**Table 1.** Quotations from Phase 1 meetings*.*

Topic	Quotation
Potential implementation challenges	“... you would need less verbal and more visual hands-on opportunities to practice skills.”
Reaching and retaining families in the program	“Once the clinicians have this tool and they are working through their regular client systems of referrals from pediatricians and family practice physicians, the awareness piece is going to be community based in the school system and then hopefully, it’s self-sustaining.”
Training issues	“Make sure that the providers feel supported, and that we have resources when we have questions in terms of the training. An easy route to get clarification, further training, or materials would be important as well.”
Reaching underserved and disadvantaged populations	“I think we need to look at faith-based organizations ... we tend to have a significant network across faith-based organizations because there is nothing else up here at times, especially for the low-income (families).”
Funding issues	“Medicaid waivers. If that’s (STEPS) something that we can get on the Medicaid Waiver list.”

### Phase 2

Phase 2 participants included 24 adolescents and young adults with autism aged 16‐29 years old and their caregivers. Mean age of participants was 21.33 (SD 4.19) years. Frequency analyses of participants showed that the sample was predominantly male (79%), White (96%), and not Hispanic or Latino (96%). Approximately 20% of the sample had a caregiver-reported previous diagnosis of intellectual disability. About 33% of the sample had a job and 43% were engaged in education or training at baseline. See Table 2 for full Phase 2 participant demographics.

**Table 2.** Participant demographics and baseline data (N=24) for Phase 2 (Hybrid Type 1 open trial).

Cognitive level	Participants (N=24)	
Intellectual disability diagnosis, n (%)		
Yes	5 (21)	
No	13 (54)	
Not Sure	6 (25)	
Sex, n (%)		
Male	19 (79)	
Female	4 (17)	
Nonbinary	1 (4)	
Race, n (%)		
Black or African American	1 (4)	
White	23 (96)	
Ethnicity, n (%)		
Hispanic or Latino	1 (4)	
Not Hispanic or Latino	23 (96)	
Annual household income (US$)		
25,000-49,999	3 (13)	
50,000-99,999	8 (33)	
Over 100,000	4 (17)	
Prefer not to answer	9 (38)	
Rehabilitation success, n (%)		
Had a job at baseline (yes)	7 (33)	
Engaged in education or training at baseline (yes)	9 (43)	
Participant age (years), mean (SD)	21.33 (4.19)	
SCQ^a^ total score, mean (SD)		
Participant	—^b^	
Caregiver	16.04 (6)	

a SCQ: Social Communication Questionnaire (total score possible range: 0‐39).

b Not available.

### Acceptability and Feasibility

Results supported acceptability of STEPS delivered in the community, evidenced by low program attrition and moderate to high program satisfaction ratings. Of the 24 participants enrolled in the STEPS trial, 22 completed the program (92% retention). Both “non-completers” dropped from the study before attending any STEPS sessions. One participant became unresponsive before scheduling sessions with their assigned therapist, and the other participant opted to withdraw before starting STEPS due to their discomfort with sessions being recorded for the study.

Participants provided ratings indicating moderate to high satisfaction on the PSS (ie, >3 on each item), suggesting that they found STEPS to be moderately helpful (mean 3.71, SD 1.26), beneficial (mean 3.47, SD 1.46), and acceptable to complete (mean 4.12, SD .99). Participants also reported moderate overall satisfaction with STEPS (mean 3.71, SD 1.21) and moderate likelihood to recommend STEPS to a peer with autism (mean 3.71, SD 1.40). Similarly, parents provided moderate to high satisfaction ratings, suggesting they found STEPS to be helpful (mean 4.19, SD .83), beneficial (mean 3.88, SD 1.03), and acceptable for their adolescent or young adult with autism to complete (mean 4.44, SD .81). They also reported high overall satisfaction with STEPS (mean 4.12, SD 1.09) and a high likelihood to recommend STEPS to other caregivers of adolescents and young adults with autism (mean 4.31, SD 1.08).

Both after they completed the initial training and after completing their STEPS cases, providers’ ratings on the AIM and IAM (ie, >3 or higher suggests moderate ratings) suggest they found STEPS acceptable as an intervention (posttraining mean 4.69, SD .63; post-STEPS cases mean 4.44, SD .66) and appropriate to their clients (posttraining mean 4.56, SD .88; post-STEPS cases mean 4.88, SD .25).

Results indicate that STEPS was feasible to implement. Fidelity ratings completed by STEPS providers indicated that 96% of STEPS treatment objectives were delivered as intended across 264 sessions. Providers delivered all objectives designated for a specific session approximately 86% of the time. Approximately 10% of sessions (n=29) were double coded for fidelity to the manual by trained research assistants. These double-coded sessions indicated that 83.19% of STEPS treatment objectives were delivered as intended. Both after they completed the initial training and after completing their STEPS cases, providers’ ratings on the FIM (ie, >3 or higher suggests moderate ratings) suggest they found STEPS feasible to implement (posttraining mean, 4.38, SD 0.32; post-STEPS cases mean, 4.94, SD 0.13). In addition, participants reported strong therapeutic alliance with their providers (mean, 50.21, SD 9.94, range 12‐60).

### Implementation

Qualitative data from the provider interviews is organized according to each CFIR domain [28]. Within Innovation (eg, the experimental program; STEPS), providers identified challenges associated with the time to prepare sessions and finding appropriate immersion activities for families in rural areas, and one provider acknowledged their own anxiety related to conducting immersion. For example, one provider commented, “It’s hard to do this [immersion] as a clinician, but for the patients, it was such an impactful part.” They also indicated they thought it could be readily used and billed for, given the CBT focus. One provider reported, “The program itself is very usable and approachable.” And another stated, “The language is respectful of the autistic community, it fits a variety of clients.” With respect to the outer setting domain (eg, the external systems), providers described the importance of family or caregiver engagement to success, and that a manualized program like STEPS could help make services more consistent for school districts. Considering the inner setting domain (eg, the setting wherein intervention is delivered), providers stated that STEPS could be used in an agency that did not specialize in autism, but that in-house expertise helped them, and that the setting must be flexible to address the diverse needs of the clients. With respect to the Individuals domain (eg, those people responsible for implementation), there was mention of helping parents find a good balance between “infantilizing” their grown child and not being involved enough; providers also indicated that the manual would be appropriate even for a clinician who does not normally work with patients with autism. For implementation (eg, those activities involved in actual implementation), several providers indicated that having strong partnerships (eg, with state vocational rehabilitation and local employers) would help, as well as ensuring that more people in the community were sensitive to their clients’ needs. One provider commented, “Having partnerships is really helpful, for example, having an external person do a mock interview for immersion. Also, it’s important to have employers on board—sensitive to clients’ needs and strengths.”

### Primary Outcomes

Although not statistically significant at the *P*=.05 level, participants reported increased transition readiness (on TRS) at end point compared to baseline *(t*_19_=−1.91; *P*=.07). Caregivers also reported significant increased transition readiness by end point for their adults with autism (*t*_15_=−4.19; *P*<.001). Clinically significant changes, operationalized as movement of >0.5 SD in TRS scores from baseline to end point, were examined. Eleven participants (55% of participants who completed end point) demonstrated clinically significant improvement on the TRS and were considered “treatment responders.” Four participants (20%) demonstrated clinically significant worsening on the TRS. Twelve caregivers (75% of caregivers who completed end point) reported that their adult or adolescent with autism had clinically significant improvement on the TRS from baseline to end point and were considered “treatment responders.” No participants significantly worsened, based on caregiver report.

On the REALS, participants and caregivers only reported increases in functional independence in a few areas, and these varied by reporter. By end point, participants reported engaging in significantly more financial tasks (*t*_14_=−2.21; *P*=.04) than at baseline. This improvement persisted six months afterward; at follow-up, participants were engaging in significantly more financial tasks *(t*_9_=−2.47; *P*=.04) compared with baseline, as well as more social *(t*_9_=−2.27; *P*=.0549) and self-care activities *(t*_9_=−2.62; *P*=.03). At the end point, caregivers reported more engagement in the community (*t*_15_=−3.31; *P*=.005) and needing less support to do so (*t*_15_=−3.21; *P*=.006) compared with baseline. At follow-up, caregivers reported a decreased need for support to apply for and get a job (*t*_7_=−12.31; *P*<.001), successfully perform at work or school (*t*_11_=−7.22; *P*<.001), and complete household tasks (*t*_13_=−4.90; *P*<.001). Caregivers also reported heightened engagement in tasks to succeed at work or school at follow-up compared with baseline (*t*_11_=−4.27; *P*=.001).

Considering the Rehabilitation Success form, 7 participants were employed at baseline. At the end point, only 4 retained their employment, and 3 additional people gained employment (n=7 at end point). By follow-up, the 4 participants employed at baseline still retained their employment, 2 of the 3 additional participants employed by end point retained their employment, and one additional participant was employed (n=7 employed at follow-up). At baseline, 9 participants were engaged in education. At the end point, 7 were still involved in education, and 6 additional participants started educational activities (n=13 at end point). At follow-up, 8 of 13 participants from the end point were still involved in education, and 2 additional participants had started educational activities (n=10 at follow-up).

### Secondary Outcomes

On the ISKS, participants did not report significantly more self-knowledge at midpoint or end point compared with baseline. On the AIR-SD, participants reported significantly increased capacity to be self-determined at both midpoint (*t*_19_=−2.52; *P*=.02) and end point (*t*_15_=−3.41; *P*=.004) compared with baseline. They did not report significant increases in opportunities to be self-determined at midpoint or end point. Caregivers reported their adults with autism having significantly more capacity to be self-determined at end point compared with baseline (*t*_15_=−3.42; *P*=.004). There were no significant differences between baseline and end point. Caregivers also reported increased opportunities for their young people with autism to be self-determined at both midpoint (*t*_17_=−3.32; *P*=.004) and end point (*t*_15_=−4.73; *P*<.001) compared with baseline.

On the SCSDSS, participants reported significantly increased beliefs that the 7 skills involved in self-determination were important for their lives by midpoint (*t*_19_=−2.16; *P*=.04) and end point (*t*_15_=−2.18; *P*=.0546) to baseline. They also reported significantly increased performance in the 7 skills involved in self-determination at both midpoint (*t*_19_=−2.15; *P*=.04) and end point (*t*_15_=−3.24; *P*=.006) compared to baseline, as well as increased overall self-determination at end point compared to baseline (*t*_15_=−2.57; *P*=.02). Caregivers similarly reported significantly increased overall self-determination at both midpoint (*t*_17_=−3.86; *P*=.001) and end point (*t*_15_=−7.48; *P*<.001) compared with baseline. They also reported increased performance in the seven skills involved in self-determination at the end point (*t*_15_=−4.64; *P*<.001). Caregivers did not report that their beliefs about the importance of the seven skills significantly changed from baseline.

On the GSES, participants reported feeling increased self-efficacy at both midpoint *(t*_19_=−2.52; *P*=.02) and end point (*t*_16_=−2.82; *P*=.01) compared with baseline. Caregivers similarly reported their young people with autism having significantly more self-efficacy at end point than baseline (*t*_15_=−7.52; *P*<.001). They did not report significant change between baseline and midpoint. On the EDI, participants reported experiencing significantly less dysphoria at end point compared to baseline (*t*_16_=3.19; *P*=.006). There was no change between participant-reported dysphoria at baseline and midpoint. Participants did not report any change in reactivity from baseline to midpoint and end point. Caregivers did not report any significant change in dysphoria or reactivity. See Table 3 for reported means of paired samples *t* tests for primary outcomes and mechanisms from baseline to end point.

**Table 3.** Phase 2 treatment outcomes at baseline to end point.

Primary outcomes	Baseline, mean (SD)	End point, mean (SD)
Participant-reported		
Transition readiness scale^a^	75.45 (15.87)	81.55 (10.18)
REALS^b^
Social activities support	−.36 (.98)	.09 (1.14)
Social activities frequency	−.46 (.79)	−.13 (.96)
Work readiness support	−.03 (.53)	.06 (.97)
Work readiness frequency	−.29 (.50)	−.22 (.73)
Work performance support	−.27 (1.14)	−.18 (.72)
Work performance frequency	−.35 (.73)	−.10 (.99)
Self-care support	−.42 (.85)	−.18 (.89)
Self-care frequency	−.49 (.63)	−.20 (1.03)
Leisure support	.06 (1.00)	.04 (1.02)
Leisure frequency	.22 (.68)	.39 (.75)
Mobility support	−.64 (1.00)	−.40 (1.05)
Mobility frequency	−.73 (.93)	−.31 (.94)
Home care support	−.01 (.70)	−.07 (.87)
Home care frequency	−.10 (.83)	−.11 (.83)
Finances support	−.42 (1.01)	−.13 (1.16)
Finances frequency	−1.42 (.96)	−.51 (1.19)^c^
Social satisfaction	.10 (.92)	.59 (.87)
Autonomy satisfaction	.25 (.85)	.61 (.92)
Work/school satisfaction	−.43 (.83)	−.29 (.86)
Caregiver-reported		
Transition readiness scale	69.44 (11.79)	81.88 (8.29)^d^
REALS
Social activities support	.29 (.85)	.43 (.81)
Social activities frequency	−.07 (.63)	.01 (.87)
Work readiness support	−.05 (.24)	.18 (.34)
Work readiness frequency	−.35 (.61)	−.32 (.61)
Work performance support	−1.20 (.51)	−.93 (.47)
Work performance frequency	−1.07 (.49)	−.71 (.39)
Self-care support	.13 (.54)	.26 (.48)
Sleep, diet, exercise support	−.07 (.72)	.03 (.93)
Leisure support	.47 (.57)	.55 (.60)
Leisure frequency	.03 (.75)	.32 (.80)
Mobility support	−.05 (.85)	.32 (.80)^c^
Mobility frequency	−.57 (.77)	−.17 (.87)^c^
Home care support	−.48 (.52)	−.29 (.39)
Home care frequency	−.80 (.72)	−.47 (.58)
Finances support	.26 (.71)	.32 (.61)
Finances frequency	−.53 (.42)	−.41 (.41)
Secondary outcomes		
Participant-Report		
ISKS^e^	27.35 (9.84)	29.53 (12.13)
AIR-SD^f^ capacity	36.38 (13.06)	42.44 (9.95)^c^
AIR-SD opportunity	41.25 (10.54)	45.38 (10.66)
SCSDSS^g^ importance	9.50 (4.16)	11.13 (3.22)^c^
SCSDSS performance	6.19 (3.83)	8.94 (4.19)^c^
SCSDSS self-determination	15.19 (6.42)	18.19 (4.65)^c^
GSES^h^	26.88 (11.45)	32.29 (13.07)^c^
EDI-dysphoria^i^	45.72 (9.27)	40.46 (6.23)
EDI-reactivity	37.81 (8.45)	35.27 (9.11)
Caregiver-Report		
AIR-SD capacity	16.19 (3.53)	19.63 (1.89)^c^
AIR-SD opportunity	42.00 (6.11)	47.69 (5.55)^d^
SCSDSS importance	12.81 (1.33)	12.44 (1.90)
SCSDSS performance	4.25 (2.14)	8.00 (3.18)^d^
SCSDSS self-determination	11.83 (2.94)	13.94 (2.01)^d^
GSES	26.50 (4.29)	32.19 (4.81)^d^
EDI dysphoria	44.48 (5.06)	41.61 (4.97)
EDI reactivity	37.44 (6.37)	34.81 (6.07)

a Transition Readiness Scale (total score; possible range: 30‐150).

b REALS: Relationships, Employment, Autonomy, and Life Satisfaction (theta scores; theta ranges differ for each domain, but within the range: -2.83‐2.40).

c *P<.05

d **P<.001

e ISKS: Integrative Self-Knowledge Scale (total score; possible range: 0‐48).

f AIR-SD: American Institutes for Research–Self-Determination Scale (domain scores; possible range for each domain: 12‐60 except for caregiver-report capacity, which is 6‐30).

g SCSDSS: Seven Component Self-Determination Skills Survey (domain scores; possible range 6‐18 for importance and performance, 7‐35 for self-determination).

h GSES: General Self-Efficacy Scale (total score; possible range: 10‐50).

i EDI: Emotion Dysregulation Inventory (T-scores; possible range: 26.10‐79.20).

## Discussion

### Principal Findings

There is a large and growing population of adults with autism who could benefit from access to evidence-based services and supports. Programs that support transition to adulthood and that address issues related to quality of life and goal attainment could have considerable impact. STEPS is such a program, previously tested in both a preliminary open trial [54] and an RCT [24].

In this hybrid type 1 open trial of STEPS delivered in a community agency by clinicians uninvolved in research, we found strong evidence for acceptability to both consumers and providers and feasibility of delivery. In terms of clinical impact, significant improvements were seen in readiness for transition and some domains of functional independence. The findings with respect to the theoretical mechanism engagement are more nuanced, showing improvement in self-determination and self-efficacy, but no significant change in self-knowledge or emotion regulation capacity. Synthesizing the qualitative interview data from the 4 providers who were unaffiliated with the research, it seems that the program could conceivably be delivered by agencies and clinicians that do not necessarily specialize in autism treatment and that having the manual as a guide was appreciated. Caregiver and family engagement in the program seemed to help with implementation and impact. Providers also reported that strong partnerships within the implementation community (eg, potential employers, job coaches, and education systems) and broader community acceptance of autism would facilitate ease of delivery.

Recent research suggests that professionals who work with transition-aged individuals with autism are concerned about their lack of knowledge about providing effective supports [55]. A readily available, manualized program could help providers target and address transition needs and provide quality services with minimal training. In fact, our findings suggest that providers found STEPS highly feasible and acceptable immediately after training, and their impressions were confirmed following delivering STEPS with high fidelity to the manual to participants with autism. STEPS, created to promote successful transition to adulthood, was initially developed with considerable input from the autism community and from experts in autism treatment [23]. In this study, we further refined the program based on community stakeholder feedback, ultimately leading to a transition support program that more readily addresses the needs of the consumers (ie, adolescents and adults with autism and their caregivers) as well as the providers. Information relevant to adoption and sustained use, across relevant implementation domains, was gleaned as well. With respect to the outer domain [28], for instance, the providers generally agreed that the program could be implemented in broad settings and by providers who do not often work with people on the spectrum.

Findings must be interpreted in the context of several considerable limitations, first and foremost being the lack of a control condition. Inferences about causation are not justified, nor can we make claims of mechanism engagement given the study design. Of course, an open trial is appropriate at this stage of research, but future implementation trials should include an active control condition and randomization. The design of the assessment battery was another limitation. One of our 2 primary outcomes, the TRS, was only collected at end point, not follow-up, which limited our ability to examine whether transition readiness persisted following completion of STEPS. It is important to note that the timing of the follow-up assessment may have impacted our objective indicators of outcome on the Rehabilitation Success measure. There were several participants who completed their follow-up over summer or holiday breaks and may have indicated they were no longer involved in educational activities despite just being on a regularly scheduled break. Related to this, STEPS is personalized and multifaceted, in that it does not target a single symptom or program. One client might be working toward obtaining a driver’s permit, another on forming their first intimate relationship, and a third on securing a full-time job. The program’s modular content is anchored to the client’s goals, such that outcomes that focus strictly on vocational status likely do not fully capture change in functioning or program impact. We also had a relatively high degree of missing data at the end point and follow-up, which restricted our ability to use other statistical methods of assessing change. A final, yet substantial, limitation is the small sample and informal qualitative analytic approach. Though arguably appropriate for the pilot of community implementation, no firm conclusions regarding the readiness of STEPS for large-scale community implementation can be made. In terms of implementation evaluation, the small sample of providers (n=4) interviewed and the stakeholder group in Phase 1 must be noted; although appropriate for a pilot study, rigorous qualitative data analysis was not viable.

Despite these limitations, this is one of the first community implementation trials to evaluate a research-supported transition program for adolescents and young adults with autism. The results suggest that further research in this area is warranted to facilitate the community implementation of effective programming that is acceptable to consumers. Programming, such as STEPS, is both needed and promising for improving adult outcomes.

### Conclusions

This study provides further support for STEPS, a program to support successful transition to adulthood for people who have autism. Prior research has shown feasibility, acceptability, and efficacy of STEPS. This community-based trial provides new evidence to support the program's ability to be delivered with integrity by practitioners, and its effectiveness clinically.

## Abbreviations

AIM: Acceptability of Implementation Measure
AIR-SD: American Institutes for Research–Self-Determination Scale
CFIR: Consolidated Framework for Implementation Research
EDI: Emotion Dysregulation Inventory
FIM: Feasibility of Implementation Measure
GSES: General Self-Efficacy Scale
IAM: Intervention Appropriateness Measure
ISKS: Integrative Self-Knowledge Scale
PSS: Program Satisfaction Scale
RCT: randomized controlled trial
REALS: Relationships, Employment, Autonomy, and Life Satisfaction
SCSDSS: Seven Component Self-Determination Skills Survey
STEPS: Stepped Transition to Employment and Postsecondary Education Success
TRS: Transition Readiness Scale
WAI-SR: Working Alliance Inventory–Short Revised
